# Supplementary material for: Chromothripsis during telomere crisis is independent of NHEJ, and consistent with a replicative origin
Source: Genome Res. 2019 May;29(5):737–49. doi: 10.1101/gr.240705.118 (PMC6499312; doi:10.1101/gr.240705.118)
Supplement: Supplemental Material [file supp_gr.240705.118_Supplemental_file_1.zip › contigs/annotated_contigs/DB45/contig.2.DB45_length_672_mean_cov_8.89136904762.docx]

**DB45_length_672_mean_cov_8.89136904762**

```
GGCTCTGCACCATGGGCCTAACCTCTATCGCAGGCATTCTCTGGGCTCCCTTGTTTGCTGGCTTTCTGTTGTATTAGCCAGGGGAAGGA
 >chr3:61182412-61182666 - E=5e-141
ACTGGATGAAGACCTGGGAACACGAGGTTGAAATATTCTTCCCTACTTTCTTCCCTGCAGCATTGTGGGTTGGCAATGGCTGTATCCCT

CTAAGGAAGGCCACAGTTCCGGTTGGGGGCCTCTCTTCAGCGGCTACAGCTTTTTCTACTGTCTGGTAACTGCCTC|AGTGC|CTCAGT
                                                                                  >chr3
GCTCAATGTTGCCCAGGCTGAAGTGCAGTGGCATGATCTCGGCTCGCTACAACCTCCACCTCCCAGCCGCCTGCCTTGGCCTCCCAAAG
:61379370-61379783 - E=9e-237
TGCCGAGATTGCAGCCTCTGCCAGGCTGCCACCCTGTCTAGGAAGTGAGGAGAGTCTCTGCCTGGCCGCCCATCCTCTGGGATGTGAGG

AGCCCTTCTGCCCAGCCGCCCAGTCTGGGAAATGAGGAGCGCCTCTAGGCGCTCACCTCCCATCTAGGAAGTGAGGAGCGTCTCTGCCC

GGCTGCCCATGGTCTGGGATGTGGGGAGTGCCTCTGCCCCGCCGCACTGTCTGAGATGTGAAGAGCGCCTCTGCCCGGCGGTGACCCCA

TCTGGGAACTGAGTAGTGTCTCTGCCCTGCCGCCACCCCGTCTGGGAGGTG
```
